# The Effect of Pomegranate Juice and Sumac Consumption in the Treatment of Outpatients with COVID-19

**DOI:** 10.1155/2022/6850342

**Published:** 2022-11-30

**Authors:** Fatemeh Forouzanfar, Mohammadreza Ahmadpoor, Mostafa Moshirian Farahi, Ali Hadianfar, Amirhossein Sahebkar, Habibollah Esmaily, Mohsen Nematy, Hassan Rakhshandeh

**Affiliations:** ^1^Neuroscience Research Center, Mashhad University of Medical Sciences, Mashhad, Iran; ^2^Department of Neuroscience, Faculty of Medicine, Mashhad University of Medical Sciences, Mashhad, Iran; ^3^Department of Persian Medicine, School of Persian and Complementary Medicine, Mashhad University of Medical Sciences, Mashhad, Iran; ^4^Pharmacological Research Center of Medicinal Plants, Mashhad University of Medical Sciences, Mashhad, Iran; ^5^Student Research Committee, Mashhad University of Medical Sciences, Mashhad, Iran; ^6^Department of Biostatistics, School of Health, Mashhad University of Medical Sciences, Mashhad, Iran; ^7^Applied Biomedical Research Center, Mashhad University of Medical Sciences, Mashhad, Iran; ^8^Biotechnology Research Center, Pharmaceutical Technology Institute, Mashhad University of Medical Sciences, Mashhad, Iran; ^9^School of Medicine, The University of Western Australia, Perth, WA 6009, Australia; ^10^Social Determinants of Health Research Center, Mashhad University of Medical Sciences, Mashhad, Iran; ^11^Department of Nutrition, Faculty of Medicine, Mashhad University of Medical Sciences, Mashhad, Iran

## Abstract

**Introduction:**

COVID-19, an epidemic of coronavirus infection, has become a major global threat. The coronavirus mainly targets the human respiratory system, followed by cytokine storm, and altered immune responses associated with disease progression and adverse outcomes. Sumac and pomegranate juice are rich in bioactive compounds, which potentially have antiviral activities. This study is aimed at investigating the effect of a diet based on the use of sumac and pomegranate juice on the treatment of outpatients with COVID-19.

**Methods:**

In this study, 182 outpatients with COVID-19 were randomly divided into two groups receiving a diet containing pomegranate juice and sumac along with standard treatment and the control group (group 2) receiving standard treatment.

**Results:**

Consumption of a diet containing pomegranate juice and sumac in outpatients with COVID-19, who were receiving standard-of-care treatment, led to a significant decrease in fever, chills, cough, weakness, smell and taste disorders, shortness of breath, diarrhea, nausea and vomiting, and abdominal pain compared with outpatients with COVID-19 who received only standard treatment.

**Conclusion:**

Clinical trials of outpatients have limitations such as patients' resilience to post-COVID-19 follow-up. However, the use of pomegranate juice and sumac can be efficacious in reducing COVID-19 symptoms. This trial is registered with IRCT20190406043175N3.

## 1. Introduction

Coronavirus Disease-2019 (COVID-19), a newly emerging respiratory disease caused by severe coronavirus 2 (SARS-CoV-2), is responsible for the recent pandemic and was started in Wuhan, China, in December 2019 [1–4]. Most patients with COVID-19 show mild to moderate symptoms, but approximately 15% develop severe pneumonia, and about 5% eventually develop acute respiratory syndrome (ARDS), septic shock, and/or multiple organ failure. The mainstay of clinical treatment includes symptom management and oxygen therapy with mechanical ventilation for patients with respiratory failure [5]. SARS-CoV-2 can damage the brain through direct neural cell infection. The inflammatory reactions, which damage neural cells, lead to brain ischemia and ensuing health problems [6]. The interaction between nutrition and the immune system is well known; therefore, any nutritional imbalance affects the integrity of the immune system. One of the most fundamental questions is the role of nutrition in the pathophysiology of COVID-19 [7]. Diet and nutrition always affect the proper functioning of the immune system and determine the risk and severity of infection [8, 9]. In addition to innate immunity, a high intake of saturated fatty acids inhibits the function of T and B lymphocytes in the acquired immune system through different mechanisms including enhancement of oxidative stress [10]. In particular, oxidative stress due to high consumption of saturated fatty acids disrupts T and B cell proliferation and maturation and causes B cell apoptosis [10]. During the COVID-19 pandemic, individuals are responsible for choosing a healthy lifestyle, eating diets rich in fruits and vegetables, exercising at leisure, maintaining a healthy weight, and getting enough sleep [11]. Medicinal plants have numerous pharmacological effects including antioxidant and anti-inflammatory activities [12, 13]. Pomegranate is a fruit with the botanical name *Punica granatum* from the family Punicaceae. It is rich in polyphenols, such as ellagitannins, gallic acid, and ellagic acid, as well as glycosylated derivatives and anthocyanins [14, 15]. The presence of these phytochemicals is directly related to their optimal, which imparts the health benefits of pomegranate, such as antiobesity, antidiabetic, and anti-inflammatory effects [16]. Fruits rich in polyphenols, such as pomegranate, have antioxidant and anti-inflammatory properties. Pomegranate has a therapeutic impact on chronic inflammatory diseases such as inflammatory bowel disease, rheumatoid arthritis, and metabolic and cardiovascular disorders. The effects of pomegranate juice against prostate cancer, diabetes, atherosclerosis, cardiovascular disease, respiratory diseases, rheumatoid arthritis, neurological diseases, and hyperlipidemia have also been reported [17, 18]. Sumac is another medicinal plant that belongs to the genus *Rhus* and has different subspecies that show slight differences based on their growing area. Sumac is commonly used as a spice and traditional remedy for centuries. The phytochemical ingredients of sumac have been extensively studied, and it has been found that this plant contains tannins, polyphenols, flavonoids, organic acids, and essential oils. Studies have shown that sumac has a protective effect against liver damage, along with its antiviral, anti-inflammatory, and antioxidant properties [19]. Thus, consumption of pomegranate juice and sumac may be an alternative strategy to overcome the symptoms of COVID-19. Given this potential, the present randomized, placebo-controlled, single-blind, parallel-group clinical trial was designed to evaluate the efficacy of pomegranate juice and sumac in patients with COVID-19.

## 2. Method

This project was carried out in Toos Health Center, Mashhad, Iran, from 8/26/1399 to 11/7/1399. Outpatients with COVID-19 have been performed. In this study, 182 eligible patients entered into either of the two groups receiving a diet containing pomegranate juice (200 ml, three times a day) and sumac (1.5 grams, two times a day) along with standard drugs or the control group (group 2) receiving treatment with standard drugs. The pomegranate juice was provided by the Sanich Company (Tehran, Iran), and sumac was provided by the Zamen Company (Mashhad, Iran).

### 2.1. Randomization Description

The numbers from 1 to 182 were written on 182 papers and placed inside a bag. Even numbers were for the intervention group, and odd numbers were for the control group. Each time a patient entered the study according to the entry criteria, a paper was removed from the bag, and the number written on the paper was the basis of assignment to each group.

### 2.2. Design

This was a randomized, placebo-controlled, single-blind, two parallel arm clinical trial.

### 2.3. Sample Size Calculation

Based on the estimated number of patients admitted to the hospital at the time of conducting the study, we initially considered that 80 patients could meet the inclusion criteria, but due to the increase in the number of patients available at the time of the study, to achieve more power, 182 patients were eventually recruited to the study.

### 2.4. Drugs Used to Treat Patients

Naproxen (500 mg tablets), hydroxychloroquine sulfate (200 mg tablets), diphenhydramine syrup, bromhexine syrup, dexamethasone, and ketorolac ampoules were also prescribed in some patients based on the physician's prescription and therapeutic guidelines.

### 2.5. Inclusion and Exclusion Criteria

[1] Patients who present with clinical symptoms consistent with COVID-19 disease and CT scan or positive PCR test for the disease and are clinically classified as an outpatient group and are referred home for treatment; [2] age under 60 years; and [3] the relative stability of cardiovascular condition. Exclusion criteria: [1] pregnant and lactating women and [2] patients who were hospitalized with COVID-19.

### 2.6. Statistical Analysis

SPSS software version 20.0 was used for statistical analysis. Categorical variables were shown as percentages and frequency. Chi-square test or Fisher's exact test was used to compare the differences between patients in two groups. To compare the proportion of patients before and after intervention in each group, we used McNemar's test. *p* values below 0.05 were considered statistically significant.

## 3. Results

### 3.1. Characteristics of the Study Groups

**Figure 1:**
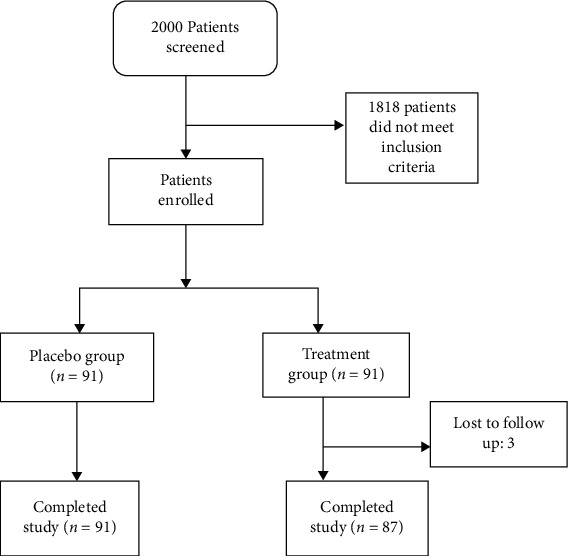
CONSORT diagram for patient recruitment and follow-up.

Figure 1 shows the patient enrollment and randomization diagram. Of the 182 patients who were randomized in this trial, 178 completed the study: 91 in the placebo group and 87 in the treatment group.

According to the chi-square test results, the two groups were not homogeneous in terms of gender frequency distribution (*p* = 0.003); therefore, the results were reported in each gender separately.

### 3.2. Respiratory Symptom

According to the results obtained before the intervention in both men and women, there was no significant difference between the two groups regarding cough and shortness of breath (*p* value > 0.05). After the intervention, there was a significant difference between the two groups regarding cough and shortness of breath in both genders (*p* value < 0.05). Moreover, according to the results of McNemar's test, there was a significant difference between the frequency of individuals in both groups in terms of cough before and after the intervention (*p* value < 0.05). In terms of shortness of breath, there was a significant difference between the frequency distribution of individuals in the pomegranate juice group in both men and women before and after the intervention (*p* value < 0.05). But there was no significant difference in the placebo group (Table 1).

### 3.3. Pain

The results showed that before the intervention in both sexes, there was no significant difference between the two treatment groups in terms of abdominal pain, muscle pain, and chest pain frequency (*p* value > 0.05) (Table 2). However, after the intervention, there was a significant difference between the two groups regarding the frequencies of abdominal pain, muscle pain, and chest pain (*p* value <0.001). According to the results of McNemar's test, in both sexes, there was a significant difference between the frequency of abdominal pain and muscle pain before and after the intervention (*p* value < 0.05).

In terms of chest pain, there was a significant difference between having chest pain before and after the intervention with pomegranate juice (*p* value < 0.05). However, in the placebo group, there was no significant difference between men and women.

In terms of headache, according to the results of Fisher's exact test, after intervention, there was no significant difference between the two groups (*p* value > 0.05). According to the results of McNemar's test, there was a significant difference between the frequency distribution of individuals in both groups in both men and women in terms of headache before and after the intervention (*p* value < 0.05) (Table 2).

### 3.4. Gastrointestinal

According to the results before the intervention in both sexes, there was no significant difference between the two treatment groups in terms of anorexia, vomiting, and diarrhea (*p* value > 0.05). After the intervention, in both sexes, there was a significant difference between the two treatment groups in terms of anorexia, vomiting, and diarrhea (*p* value < 0.001) (Table 3). According to the results of McNemar's test, there was a significant difference between the frequency distribution of people of both sexes in the pomegranate juice group in terms of having anorexia before and after the intervention (*p* value < 0.05). However, there was no significant difference in the placebo group.

There was a significant difference between women in terms of vomiting before and after the intervention (*p* value < 0.05). There was a significant difference between having diarrhea before and after the intervention in both men groups (*p* value < 0.05). However, there was no significant difference in women in the placebo group (Table 3).

### 3.5. General Symptoms

According to the results before the intervention, in both men and women, there was no significant difference between the two groups in terms of fever, sore throat, taste and smell disorders, and chills (*p* value > 0.05). After the intervention, according to the results of Fisher's exact test, there was no significant difference between the two treatment groups in terms of fever (*p* value > 0.05) (Table 4). Moreover, according to the results of McNemar's test, there was a significant difference between the frequency distribution of individuals in both groups by gender in terms of having a fever before and after the intervention (*p* value < 0.001). After the intervention, according to Fisher's exact test results, there was a significant difference between the two groups in terms of sore throat (*p* value < 0.05). Moreover, according to the results of McNemar's test, there was a significant difference between the frequency distribution of individuals in both groups by gender in terms of having a sore throat before and after the intervention (*p* value < 0.05). After the intervention, according to Fisher's exact test results, there was no significant difference between the two groups in terms of taste and smell disorders (*p* value > 0.05). Moreover, according to the results of McNemar's test, there was a significant difference between the frequency distribution of men in the drug group in terms of taste and smell disorders before and after the intervention (*p* value < 0.05), but in women, there was no significant difference between the groups in the above-mentioned complications. After the intervention, according to Fisher's exact test results, there was no significant difference between the two treatment groups in terms of chills (*p* value > 0.05). According to the results of McNemar's test, there was a significant difference between the frequency distribution of individuals in both groups in both men and women in terms of chills before and after the intervention (*p* value < 0.05). According to the results obtained before the intervention in both sexes, there was a significant difference between the two treatment groups in terms of weakness before the intervention (*p* value < 0.05). After the intervention, there was a significant difference between the two treatment groups in terms of weakness (*p* value < 0.05). According to the results of McNemar's test, there was a significant difference in the frequency distribution of individuals in the pomegranate juice group, both in men and women, in terms of weakness before and after the intervention (*p* value < 0.05). However, there was no significant difference in the placebo group. According to the results obtained before the intervention in both sexes, there was a significant difference between the two treatment groups regarding dizziness before the intervention (*p* value < 0.05). After the intervention, in women, there was a significant difference between the two groups in terms of dizziness (*p* value < 0.05), but there was no significant difference in men. According to the results of McNemar's test, there was a significant difference between the frequency distribution of individuals in both groups in both men and women in terms of dizziness before and after the intervention (*p* value < 0.05) (Table 4).

## 4. Discussion

Given the vital role of the human immune system in preventing and combating the virus, the best and easiest way is to boost the immune system by providing enough fluids, electrolytes, proteins, and energy. This study is aimed at evaluating the efficacy of a dietary intervention in COVID-19 outpatients. The most common symptoms of COVID-19 are fever, cough, and shortness of breath. Some less common symptoms in patients include anorexia, diarrhea, abdominal pain, dizziness, headache, impaired consciousness, acute cerebrovascular disease, ataxia, neuralgia, and fatigue [20, 21]. Pomegranate produces a relatively reddish purple juice that contains an average of 85.4% water and 15.6% dry matter, including sugars, organic acids, pectins, anthocyanins, polyphenols, vitamins, and minerals [22]. However, water content and organoleptic properties are strongly associated with pomegranate diversity and pomegranate juice production technology [22]. The content of soluble polyphenols in pomegranate juice varies in the range of 0.2 to 1.0% depending on its type, including hydrolyzable tannins, ellagic acid derivatives, and flavonoids. Punicalagin, a large polyphenol, belongs to the ellagitannins family that is responsible for more than half of the antioxidant effect of pomegranate juice. Pomegranate juice is an essential rich source of flavonoids, including flavonols (catechins, epicatechins, and gallocatechin) and anthocyanins. Anthocyanins are water-soluble plant pigments responsible for the red color of fruits and juices. They include 3-glucosides and 3,5-glucosides of delphinidin, cyanidin, and pelargonidin [23]. Phenolic acids include hydroxybenzoic acids (mainly gallic acid and ellagic acid) and hydroxycinnamic acids, mainly caffeic acid, chlorogenic acid, and p-coumaric acid. Other chemical compounds in pomegranate juice include sugars (glucose, fructose, and sucrose), organic acids (citric acid, malic acid, tartaric acid, fumaric acid, succinic acid, ascorbic acid, etc.), amino acids (proline, valine, methionine, glutamic acid, and aspartic acid), indoleamine (tryptamine, serotonin, and melatonin), tocopherols, and minerals (Fe, Ca, Cl, Cu, K, Mg, Mn, Na, Sn, and Zn) [23]. Ellagitannins are associated with the prebiotic potential and antimicrobial activity of fruit juices. Laboratory studies have proven the potential of pomegranate extract as an antitumor agent against various cancers, including prostate cancer, renal cell carcinoma, papillary thyroid carcinoma cells, and cervical cancer cell lines [23]. Pomegranate polyphenols in extracts, juices, or as isolated compounds have excellent antiviral activities against herpes simplex types 1 and 2 (HSV-1 and HSV-2), influenza viruses (H1N1, H3N2, and H5N1), human immunodeficiency virus-1 (HIV-1, clades A to G, and group O), HIV-2, human enterovirus 71 (EV71), hepatitis C virus (HCV), adenoviruses, rotaviruses, feline calicivirus (FCV-F9), mosquito-borne dengue virus (DENV), and norovirus (MNV-1). Polyphenols have antiviral effects on host cells through various mechanisms such as structural damage to the virion and inactivation of the virus, inhibition of viral polymerase activity, protein expression, and RNA replication, or blockade of absorption of the virus on host cells [24]. The antioxidant capacity of pomegranate juice is three times those of red wine and green tea based on the evaluation of the free radical scavenging capacity. It was also shown that the level of antioxidants is significantly higher than ordinary fruit juices such as grape, cranberry, grapefruit, or orange juice. The primary antioxidant polyphenols in pomegranate juice include ellagitannins and anthocyanins. Ellagitannins account for 92% of the antioxidant activity of pomegranate juice and are concentrated in the fruit's peel, membranes, and piths [25]. Punicalagins are the main ellagitannins in the whole fruit and can be hydrolyzed to ellagic acid and other smaller polyphenols in the body. This metabolism depends on the fruit cultivar, processing, and storage conditions [25].

The ingredients of sumac are tannins, flavonoids, anthocyanins, isoflavones, terpenoids, and diterpenes [26]. A study was performed on the chemical properties of sumac fruits, which contained 2.6% protein, 7.4% fat, 14.6% fiber, and 1.8% ash [26]. The most abundant phenolic compound in sumac fruits is gallic acid. The main vitamins in sumac are thiamine (B1), riboflavin (B2), pyridoxine (B6), cyanocobalamin (B12), nicotinamide, biotin, and ascorbic acid. Among these vitamins, pyridoxine was the most abundant, followed by ascorbic acid, thiamine, and riboflavin [26]. There have been studies suggesting that sumac reduces elevated C-reactive protein levels in diabetes, cancer, and atherosclerosis [27]. In previous studies, sumac has also shown inhibitory activity against respiratory (influenza A, influenza B, and measles) and herpes (HSV-1, HSV-2, and varicella zoster virus (VZV)) and HIV viruses [19].

## 5. Conclusion

Consumption of pomegranate juice (200 ml, three times a day) and sumac (1.5 grams, two times a day) in outpatients with COVID-19 reduced fever, chills, cough, smell and taste disorders, shortness of breath, diarrhea, nausea, vomiting, and abdominal pain compared to outpatients who received only standard treatment.

## Figures and Tables

**Table 1:** Frequency of respiratory symptoms in the two groups before and after intervention.

Respiratory symptom			Before intervention		After intervention		McNemar's test
			Pomegranate juice+sumac	Placebo	Pomegranate juice+sumac	Placebo	
			**N** (%)	**N** (%)	**N** (%)	**N** (%)	
Cough	Men	Yes	66 (97.1)	48 (92.3)	18 (26.5)	38 (47.5)	^#^ *p* < 0.001 ^∗^*p* = 0.01
		No	2 (2.9)	4 (7.7)	50 (73.5)	13 (25.5)	
		Chi-square test	*p* = 0.43		*p* < 0.001		—
	Women	Yes	19 (100.0)	39 (100.0)	4 (21.1)	20 (52.6)	^#^ *p* < 0.001 ^∗^*p* < 0.001
		No	0 (0.0)	0 (0.0)	15 (78.9)	18 (47.4)	
		Chi-square test	*p* > 0.99		*p* = 0.03		—

Shortness of breath	Men	Yes	46 (67.6)	30 (57.7)	2 (2.9)	27 (52.9)	^#^ *p* < 0.001 ^∗^*p* = 0.24
		No	22 (32.4)	22 (42.3)	66 (97.1)	24 (47.1)	
		Chi-square test	*p* = 0.34		*p* < 0.001		—
	Women	Yes	12 (63.20)	31 (79.5)	0 (0.0)	28 (37.7)	^#^ *p* < 0.001 ^∗^*p* = 0.58
		No	7 (36.8)	8 (20.5)	19 (100.0)	10 (26.3)	
		Chi-square test	*p* = 0.2		*p* < 0.001		—

^#^Pomegranate juice+sumac. ^∗^Placebo.

**Table 2:** Frequency of pain symptoms in the two groups before and after intervention.

Pain			Before intervention		After intervention		McNemar's test
			Pomegranate juice+sumac	Placebo	Pomegranate juice+sumac	Placebo	
			**N** (%)	**N** (%)	**N** (%)	**N** (%)	
Abdominal pain	Men	Yes	63 (92.6)	42 (80.8)	1 (1.5)	23 (45.1)	^#^ *p* < 0.001 ^∗^*p* = 0.001
		No	5 (7.4)	10 (19.2)	67 (98.5)	28 (54.9)	
		Chi-square test	*p* = 0.051		*p* < 0.001		—
	Women	Yes	17 (89.5)	32 (82.1)	0 (0.0)	21 (55.3)	^#^ *p* < 0.001 ^∗^*p* = 0.006
		No	2 (10.5)	7 (17.9)	19 (100.0)	17 (44.7)	
		Chi-square test	*p* = 0.7		*p* < 0.001		—

Muscle pain	Men	Yes	68 (100.0)	5 (100.0)	1 (1.5)	35 (86.6)	^#^ *p* < 0.001 ^∗^*p* < 0.001
		No	0 (0.0)	0 (0.0)	67 (98.5)	16 (31.4)	
		Chi-square test	*p* > 0.99		*p* < 0.001		—
	Women	Yes	19 (100.0)	3 (100.0)	0 (0.0)	31 (81.6)	^#^ *p* < 0.001 ^∗^*p* = 0.02
		No	0 (0.0)	0 (0.0)	19 (100.0)	7 (18.4)	
		Chi-square test	*p* > 0.99		*p* < 0.001		—

Chest pain	Men	Yes	66 (97.1)	5 (100.0)	2 (2.9)	47 (92.20)	^#^ *p* < 0.001 ^∗^*p* = 0.13
		No	2 (2.9)	0 (0.0)	66 (97.1)	4 (7.8)	
		Chi-square test	*p* = 0.5		*p* < 0.001		—
	Women	Yes	19 (100.0)	39 (100.0)	0 (0.0)	38 (100.0)	^#^ *p* < 0.001 ^∗^*p* = 0.99
		No	0 (0.0)	0 (0.0)	19 (100.0)	0 (0.0)	
		Chi-square test	*p* > 0.99		*p* < 0.001		—

Headache	Men	Yes	67 (98.5)	45 (86.5)	0 (0.0)	11 (21.6)	^#^ *p* < 0.001 ^∗^*p* < 0.001
		No	1 (1.5)	7 (13.5)	68 (100.0)	40 (78.4)	
		Chi-square test	*p* = 0.02		*p* = 0.42		—
	Women	Yes	19 (100.0)	36 (92.3)	1 (5.3)	6 (15.8)	^#^ *p* < 0.001 ^∗^*p* < 0.001
		No	0 (0.0)	3 (7.7)	18 (100.0)	32 (84.2)	
		Chi-square test	*p* = 0.11		*p* = 0.25		—

^#^Pomegranate juice. ^∗^Placebo.

**Table 3:** Frequency of gastrointestinal symptoms in the two groups before and after intervention.

Gastrointestinal			Before intervention		After intervention		McNemar's test
			Pomegranate juice+sumac	Placebo	Pomegranate juice+sumac	Placebo	
			**N** (%)	**N** (%)	**N** (%)	**N** (%)	
Anorexia	Men	Yes	66 (97.1)	5 (100.0)	2 (2.9)	2 (3.9)	^#^ *p* < 0.001 ^∗^*p* = 0.5
		No	2 (2.9)	0 (0.0)	66 (97.1)	2 (3.9)	
		Chi-square test	*p* = 0.5		*p* < 0.001		—
	Women	Yes	18 (94.7)	3 (100.0)	1 (5.3)	38 (100.0)	^#^ *p* < 0.001 ^∗^*p* = 0.99
		No	0 (0.0)	0 (0.0)	18 (94.7)	0 (0.0)	
		Chi-square test	*p* = 0.13		*p* < 0.001		—

Vomiting	Men	Yes	62 (91.2)	46 (88.5)	1 (1.5)	20 (39.2)	^#^ *p* < 0.001 ^∗^*p* < 0.001
		No	6 (8.8)	6 (11.5)	67 (98.5)	31 (60.8)	
		Chi-square test	*p* = 0.6		*p* < 0.001		—
	Women	Yes	17 (89.5)	33 (84.6)	0 (0.0)	15 (39.5)	^#^ *p* < 0.001 ^∗^*p* < 0.001
		No	2 (10.5)	6 (15.4)	19 (100.0)	23 (60.5)	
		Chi-square test	*p* = 0.9		*p* < 0.001		—

Diarrhea	Men	Yes	57 (83.8)	43 (82.7)	1 (1.5)	32 (62.7)	^#^ *p* < 0.001 ^∗^*p* = 0.02
		No	11 (16.2)	9 (17.3)	67 (98.5)	19 (37.3)	
		Chi-square test	*p* = 0.8		*p* < 0.001		—
	Women	Yes	17 (89.5)	33 (84.6)	0 (0.0)	25 (65.8)	^#^ *p* < 0.001 ^∗^*p* = 0.14
		No	2 (10.5)	6 (15.4)	19 (100.0)	13 (34.0)	
		Chi-square test	*p* = 0.9		*p* < 0.001		—

^#^Pomegranate juice. ^∗^Placebo.

**Table 4:** Frequency of general symptoms in the two groups before and after intervention.

General symptoms			Before intervention		After intervention		McNemar's test
			Pomegranate juice+sumac	Placebo	Pomegranate juice+sumac	Placebo	
			**N** (%)	**N** (%)	**N** (%)	**N** (%)	
Fever	Men	Yes	51 (75.0)	22 (42.3)	0 (0.0)	1 (2.0)	^#^ *p* < 0.001 ^∗^*p* < 0.001
		No	17 (25.0)	30 (57.7)	68 (100.0)	58 (98.0)	
		Chi-square test	*p* = 0.05		*p* = 0.43		—
	Women	Yes	11 (57.9)	29 (74.4)	0 (0.0)	0 (0.0)	^#^ *p* = 0.001 ^∗^*p* < 0.001
		No	8 (42.1)	10 (25.6)	19 (100.0)	38 (100.0)	
		Chi-square test	*p* = 0.2		*p* > 0.99		—

Sore throat	Men	Yes	63 (92.6)	5 (100.0)	3 (4.4)	15 (29.4)	^#^ *p* < 0.001 ^∗^*p* = 0.01
		No	5 (7.4)	0 (0.0)	65 (95.6)	36 (70.6)	
		Chi-square test	*p* = 0.07		*p* < 0.001		—
	Women	Yes	18 (94.7)	3 (100.0)	1 (5.3)	12 (31.6)	^#^ *p* < 0.001 ^∗^*p* < 0.001
		No	1 (5.3)	0 (0.0)	18 (94.7)	26 (68.4)	
		Chi-square test	*p* = 0.3		*p* = 0.4		—

Smell and taste disorders	Men	Yes	38 (55.9)	25 (48.1)	17 (25.0)	21 (41.2)	^#^ *p* < 0.001 ^∗^*p* = 0.54
		No	30 (44.1)	27 (51.9)	51 (75)	30 (58.8)	
		Chi-square test	*p* = 0.4		*p* = 0.07		—
	Women	Yes	13 (68.4)	17 (43.6)	8 (42.1)	20 (52.6)	^#^ *p* = 0.06 ^∗^*p* = 0.99
		No	6 (31.6)	22 (56.4)	11 (57.9)	18 (47.4)	
		Chi-square test	*p* = 0.09		*p* = 0.8		—

Chills	Men	Yes	61 (89.7)	50 (96.2)	0 (0.0)	1 (2.0)	^#^ *p* < 0.001 ^∗^*p* < 0.001
		No	7 (10.3)	2 (3.8)	68 (100.0)	50 (98.0)	
		Chi-square test	*p* = 0.16		*p* = 0.42		
	Women	Yes	16 (84.2)	38 (97.4)	0 (0.0)	1 (2.6)	^#^ *p* < 0.001 ^∗^*p* < 0.001
		No	3 (25.8)	1 (2.6)	19 (100.0)	37 (97.4)	
		Chi-square test	*p* = 0.06		*p* = 0.99		—

Weakness	Men	Yes	58 (85.3)	5 (100.0)	8 (11.8)	50 (98.0)	^#^ *p* < 0.001 ^∗^*p* = 0.99
		No	10 (14.7)	0 (0.0)	60 (88.2)	1 (2.0)	
			*p* = 0.001		*p* < 0.001		—
	Women	Yes	15 (78.9)	3 (100.0)	3 (15.8)	38 (100.0)	^#^ *p* = 0.002 ^∗^*p* = 0.99
		No	4 (21.2)	0 (0.0)	16 (84.2)	0 (0.0)	
		Chi-square test	*p* = 0.09		*p* < 0.001		—

Dizziness	Men	Yes	55 (80.9)	33 (63.5)	1 (1.5)	3 (5.9)	^#^ *p* < 0.001 ^∗^*p* < 0.001
		No	13 (19.1)	19 (36.5)	67 (98.5)	48 (94.1)	
		Chi-square test	*p* = 0.03		*p* = 0.3		—
	Women	Yes	17 (8.5)	23 (59.0)	0 (0.0)	0 (0.0)	*p* < 0.001 ^∗^*p* < 0.001
		No	2 (10.5)	16 (41.0)	19 (100.0)	3 (100.0)	
		Chi-square test	*p* = 0.03		*p* < 0.001		—

^#^Pomegranate juice+sumac. ^∗^Placebo.

## Data Availability

The datasets of the current study are available from the corresponding author on request.
